# Success of prophylactic antiviral therapy for SARS-CoV-2: Predicted critical efficacies and impact of different drug-specific mechanisms of action

**DOI:** 10.1371/journal.pcbi.1008752

**Published:** 2021-03-01

**Authors:** Peter Czuppon, Florence Débarre, Antonio Gonçalves, Olivier Tenaillon, Alan S. Perelson, Jérémie Guedj, François Blanquart

**Affiliations:** 1 Institute of Ecology and Environmental Sciences of Paris, Sorbonne Université, CNRS, UPEC, IRD, INRAE, Paris, France; 2 Center for Interdisciplinary Research in Biology, CNRS, Collège de France, PSL Research University, Paris, France; 3 Université de Paris, INSERM, IAME, Paris, France; 4 Theoretical Biology and Biophysics, Los Alamos National Laboratory, Los Alamos, New Mexico, United States of America; 5 New Mexico Consortium, Los Alamos, New Mexico, United States of America; University of Pittsburgh, UNITED STATES

## Abstract

Repurposed drugs that are safe and immediately available constitute a first line of defense against new viral infections. Despite limited antiviral activity against SARS-CoV-2, several drugs are being tested as medication or as prophylaxis to prevent infection. Using a stochastic model of early phase infection, we evaluate the success of prophylactic treatment with different drug types to prevent viral infection. We find that there exists a critical efficacy that a treatment must reach in order to block viral establishment. Treatment by a combination of drugs reduces the critical efficacy, most effectively by the combination of a drug blocking viral entry into cells and a drug increasing viral clearance. Below the critical efficacy, the risk of infection can nonetheless be reduced. Drugs blocking viral entry into cells or enhancing viral clearance reduce the risk of infection more than drugs that reduce viral production in infected cells. The larger the initial inoculum of infectious virus, the less likely is prevention of an infection. In our model, we find that as long as the viral inoculum is smaller than 10 infectious virus particles, viral infection can be prevented almost certainly with drugs of 90% efficacy (or more). Even when a viral infection cannot be prevented, antivirals delay the time to detectable viral loads. The largest delay of viral infection is achieved by drugs reducing viral production in infected cells. A delay of virus infection flattens the within-host viral dynamic curve, possibly reducing transmission and symptom severity. Thus, antiviral prophylaxis, even with reduced efficacy, could be efficiently used to prevent or alleviate infection in people at high risk.

## Author summary

Antiviral therapy taken prophylactically can prevent a viral infection. Administering antiviral drugs in prophylaxis to health care workers or other people at risk could be especially important in the SARS-CoV-2 pandemic. Monoclonal antibodies against the SARS-CoV-2 spike protein and small molecule antiviral drugs could be used for pre- or post-exposure prophylaxis. We predict that combination therapy with two drugs with different modes of action and enough efficacy have the potential to fully prevent SARS-CoV-2 infection. We provide a prediction for the critical combination of drug efficacies above which viral establishment is suppressed entirely. Prophylactic antiviral therapy could be feasible, efficient, and alleviate the burden on healthcare systems.

## Introduction

The novel coronavirus SARS-CoV-2 rapidly spread around the globe in early 2020 [1–4]. As of January 12^th^ 2021, more than 91 million cases and 1.9 million associated deaths have been detected worldwide [5]. SARS-CoV-2 causes substantial morbidity and mortality with about 4% of cases being hospitalized overall, but up to 47% in the oldest age group [6–8], and a case fatality ratio of the order of 1% overall, which is again much higher in the elderly [6, 9, 10]. With a short epidemic doubling time of 2 to 7 days when uncontrolled [1, 7, 11], this epidemic can rapidly overburden healthcare systems [12]. Many countries have imposed social distancing measures to reduce incidence. Lifting these measures while keeping the epidemic in check may require a combination of intensive testing, social isolation of positive cases, efficient contact tracing and isolation of contacts [13, 14]. Even if these measures are locally successful in keeping the disease at low prevalence, the presence of SARS-CoV-2 in many countries and substantial pre-symptomatic transmission [14, 15] suggest that the virus may continue to circulate for years to come.

Existing antiviral therapies can be repurposed to treat COVID-19 in infected individuals [16–18]. Clinical trials to test several agents are underway, but existing antivirals have limited efficacy against SARS-CoV-2 and are most efficient in reducing viral load when taken early in infection [19–21]. Prophylactic therapy using (repurposed) antivirals has been proposed [22–24], is currently being tested [25] (e.g. study NCT04497987), and is successfully used in the prevention of HIV infection and malaria [26, 27]. Monoclonal antibodies, such as REGN-COV2 and Eli Lilly’s bamlanivimab, both authorized for emergency use in the United States as of January 7^th^ 2021 [28], could also be used for prophylaxis. These agents could be an essential tool to reduce the probability of SARS-CoV-2 infection in individuals at high risk, e.g. the elderly (especially those in nursing homes), individuals with co-morbidities, and health care workers, thus substantially reducing the burden on health care systems. Depending on the safety profile of the antiviral drug, it could be taken pre-exposure or just after contact with an infected individual (post-exposure). In this study, we integrate recent knowledge on SARS-CoV-2 host-pathogen interactions and the mechanisms of action of the antivirals currently tested in clinical trials to evaluate the efficacy of prophylactic antiviral therapy. We calculate the probability of establishment of an infection for a given viral inoculum in an individual under prophylactic antiviral therapy.

## Results

### Within-host model of viral dynamics

We consider a stochastic analog of a standard target-cell-limited model for viral kinetics. In this model, infectious virus particles, *V_I_*, infect target cells, *T*, i.e. cells susceptible to infection, in the upper respiratory tract at rate *β*. Initially, the resulting infected cells, *I*_1_, do not produce virus and are said to be in the eclipse phase of infection. After an average duration 1/*k*, these cells exit the eclipse phase and become productively infected cells, *I*_2_, which continuously produce virus at rate *p* per cell. A fraction *η* of these virions is infectious (*V*_*I*_) and can potentially infect new target cells (*T*); the remainder of the produced virions, (1 − *η*), is non-infectious, denoted *V*_*NI*_. Non-infectious virions may be the result of deleterious mutations, or misassembly of the virus particle. Free virions (of both types) and infected cells are lost with rate *c* and *δ*, respectively. A potential early humoral immune response could contribute to the clearance parameter *c* or reduce the infection rate *β*. In other models, the innate immune response was assumed to increase the infected cell death rate *δ* [21] or to reduce the number of available target cells by putting them into a refractory state [19, 29]. It is currently not possible to decide on the best model structure to describe innate immunity given the limited available data during early infection. For large numbers of target cells, infected cells and virions, the following set of differential equations describes the dynamics:

$$\begin{array}{c} \begin{array}{cc} \frac{dT}{dt} & =-\beta TV_{I},\\ \frac{dI_{1}}{dt} & =\beta TV_{I}-kI_{1},\\ \frac{dI_{2}}{dt} & =kI_{1}-\delta I_{2},\\ \frac{dV_{I}}{dt} & =\eta pI_{2}-cV_{I}-\beta TV_{I},\\ \frac{dV_{NI}}{dt} & =\left(1-\eta \right)pI_{2}-cV_{NI}. \end{array} \end{array}$$ (1)

**Fig 1:**
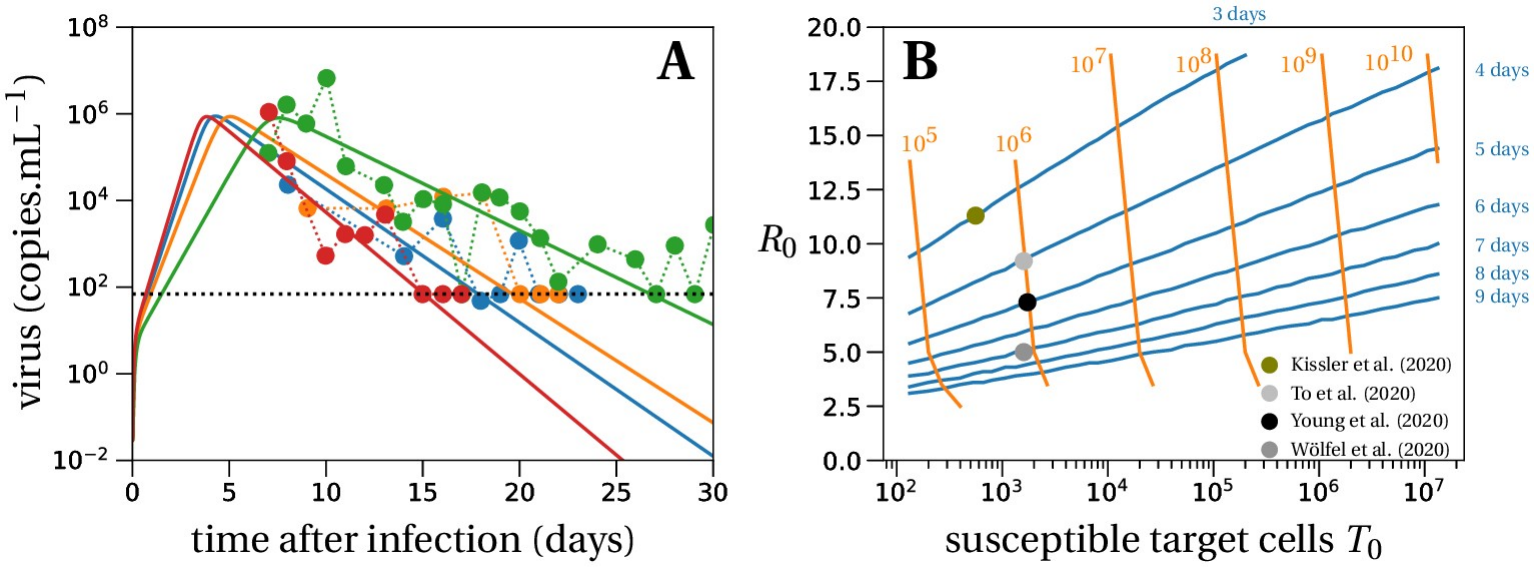
Deterministic within-host dynamics of SARS-CoV-2. (A) Model predictions using the target cell-limited model in four typical patients of ref. [31]. The estimated mean for the within-host *R*_0_ of all patients from ref. [31] is 7.69. Parameter values are given in Table B in S1 Text. The dotted line depicts the detection threshold. (B) We plot the contour lines of the viral peak time (blue lines) and the number of virus particles at the viral peak per mL (orange lines) as a function of *R*_0_ and the number of susceptible target cells *T*_0_. The lines are obtained by evaluating the set of differential equations in Eq (1) with different values of *T*_0_ (x-axis) and *R*_0_ (y-axis). The initial amount of virus particles per mL, *V*_*I*_(0) = 1/30, corresponds to 1 infectious virus particle in absolute numbers in the total upper respiratory tract, which we assume has a volume of 30 mL. The contour lines for viral loads (orange) stop if the viral peak is reached later than 20 days after infection, which can happen for low values of the within-host *R*_0_. The parameters of the model are set to: *k* = 5 day^−1^, *c* = 10 day^−1^, *δ* = 0.595 day^−1^, *p* = 11, 200 day^−1^, *η* = 0.001 and *β* = *cδR*_0_/(*T*_0_(*ηp* − *δR*_0_)) day^−1^. Dots depict averages of some data sets from Table 1.

To generate parameter estimates for system (1), we followed the methodology of a previous study (Section G in S1 Text) [19]. We show examples of our predictions in four out of 13 analyzed patients (Fig 1A). An important quantity in determining the dynamics of this model is the within-host basic reproductive number *R*_0_. It reflects the mean number of secondary cell infections caused by a single infected cell at the beginning of the infection when target cells are not limiting. Using next-generation tools for invasion analysis [30], the within-host basic reproductive number for model (1) is given by

$$\begin{array}{c} R_{0}=\frac{\beta T_{0}}{c+\beta T_{0}}\frac{\eta p}{\delta }, \end{array}$$ (2)

where *T*_0_ is the initial number of target cells. *R*_0_ is the product of two terms: *βT*_0_/(*c* + *βT*_0_), which corresponds to the probability that the virus infects a cell before it is cleared, and *ηp*/*δ*, which is the mean number of infectious virus particles produced by an infected cell during its lifespan of average duration 1/*δ*. The mean number of overall virions produced, both infectious and non-infectious, is called the “burst size” (*N* = *p*/*δ*).

We study the within-host dynamics of SARS-CoV-2 in the early stage of an infection, when the number of infected cells is small and stochastic effects are important. To do so, we define a set of reactions corresponding to the differential equations in (1) [32, 33]:

$$\begin{array}{c} \begin{array}{cccccc} & V_{I}+T & & \overset{\beta }{⟶}I_{1}, & & \;\text{infection}\;\text{of}\;\text{target}\;\text{cells},\\ & I_{1} & & \overset{k}{⟶}I_{2}, & & \;\text{end}\;\text{of}\;\text{eclipse}\;\text{phase},\\ & I_{2} & & \overset{\delta }{⟶}⌀, & & \;\text{infected}\;\text{cell}\;\text{death},\\ & I_{2} & & \overset{\eta p}{⟶}I_{2}+V_{I}, & & \;\text{infectious}\;\text{virus}\;\text{production},\\ & I_{2} & & \overset{(1-\eta )p}{⟶}I_{2}+V_{NI}, & & \;\text{non-infectious}\;\text{virus}\;\text{production},\\ & V_{I},V_{NI} & & \overset{c}{⟶}⌀, & & \;\text{virus}\;\text{clearance}. \end{array} \end{array}$$ (3)

Because we are interested in early events, we subsequently assume in the analysis that the number of target cells remains equal to *T*_0_ (see Section A in S1 Text). This is a reasonable assumption as long as the number of infectious virions is much smaller than the number of target cells (*V*_*I*_(*t*) ≪ *T*(*t*)).

### Parameterization of the model

The exact values of the within-host basic reproductive number *R*_0_ and the burst size *N* are critical to our predictions. Based on data from 13 patients [31] with an observed peak viral load of order 10^6^ virions per mL, we estimate the within-host basic reproductive number to be *R*_0_ = 7.69 with the 90% confidence interval being (1.43,13.95), cf. Section G in S1 Text for more details. In ref. [19] a sensitivity analysis of the same model without distinction of infectious and non-infectious virus was conducted. This sensitivity analysis revealed that the 95% confidence interval of the within-host *R*_0_ is (1.9,17.6), in line with other estimates of *R*_0_ for SARS-CoV-2 in the upper respiratory tract [34]. To further explore the range of *R*_0_ values compatible with other available data sets, we systematically solved the system of Eq (1) and examined the peak viral load and the time when the peak is reached, as a function of the number of susceptible target cells *T*_0_ and *R*_0_, with all other parameters held constant at values given in Fig 1B. For peak viral loads between 10^5^ and 10^8^ copies per mL and peak timing between 3 and 9 days, encompassing the range of average outcomes observed in multiple studies (Table 1), *R*_0_ may vary between 3 and 13 (Fig 1B). We note that there is substantial inter-individual variability in viral loads, and some patients present an observed peak viral load at 10^9^ copies/mL or higher [35, 36], compatible with a *R*_0_ of 15 or more. The mean observed peak viral load across the studies surveyed was 10^6^ copies/mL (Table 1).

**Table 1:** Literature review of SARS-CoV-2 viral load trajectories within hosts.

Country / Setting	# ind.	Mean observed peak viral load [copies.mL^−1^]	Mean time of observed viral peak [days after infection]	Reference
Singapore / hospital / nasopharyngeal swabs	13	10^6^ (max. 3 × 10^8^)	5-10 (a few days after symptoms)	[31]
Germany / hospital / nasopharyngeal swabs	9	7 ×1 0^5^ (max. 2 × 10^9^)	≤ 7 (already declining at admission)	[37]
mainland China / throat swabs	67	10^5^ (max. 3 × 10^7^)	≤ 5 (no increase after symptom onset)	[38]
mainland China / throat swabs	94	10^5^ (max. 7 × 10^8^)	5	[39]
Hong Kong / hospital / throat swabs	23	10^6^ (max. 3 × 10^7^)	4	[40]
France / hospital / nasopharyngeal swabs	25	6 × 10^8^ (max. 2 × 10^11^)	9 (inferred in prospective study)	[41]
USA / NBA players and staff / nasopharyngeal and throat swabs	68	4 × 10^5^ (max. 10^7^)	3	[36]*

Alongside the mean observed peak viral loads, we also state the maximal peak viral loads from the cited studies (minimal values are not always provided in the references). These maximal values inform about the plausible upper bound for the within-host reproductive number *R*_0_.

*Cycle threshold (Ct) values are reported. Conversion to viral loads is according to personal communication with David Ho (Columbia University).

The burst size for SARS-CoV-2 is unknown. Estimates of the burst size for other coronaviruses range from 10 − 100 [42] to 600 − 700 [43, 44] infectious virions. We assume that the proportion of infectious virions produced by an infected cell is *η* = 10^−3^. This value is motivated by the fraction of infectious virus in an inoculum injected into rhesus macaques, *η* = 1.33 × 10^−3^ [45]. The total viral burst size is then between 10, 000 and 100, 000 virions. Such large total burst size is suggested by electron microscopy showing the emergence of huge numbers of virions from cells infected by SARS-CoV-1 [46, 47] (see also [48], a webpage dedicated to SARS-CoV-2: e.g. https://www.flickr.com/photos/niaid/49557785797/in/album-72157712914621487/). Given the uncertainty in this parameter, we ran simulations with a small (parameter set ‘LowN’) and a large burst size (parameter set ‘HighN’). The exact values of the LowN and HighN parameter sets are given in Table 2.

**Table 2:** Model parameters used in the stochastic simulations.

Parameter set	*ηp* [day^−1^]	*T*_0_ [cells]	*ηN* [virions]	*R*_0_ [cells]
LowN	11.2	4 × 10^4^	18.8	7.69
HighN	112	4 × 10^3^	188	7.69

Parameters not shown in the table are not changed between the simulations and are set to: *k* = 5 day^−1^, *δ* = 0.595 day^−1^, *c* = 10 day^−1^, *η* = 10^−3^, *β* = *cδR*_0_/(*T*_0_(*ηp* − *δR*_0_)) day^−1^.

### Survival and establishment of the virus within the host

As shown previously [32, 33], with the model dynamics defined in (3) the probability that a viral inoculum of size *V*_0_ establishes an infection within the host is given by:

$$\begin{array}{c} \varphi =\{\begin{array}{cc} 1-{\left(1-\frac{R_{0}-1}{\eta N}\right)}^{V_{I}\left(0\right)}, & \text{if}\;R_{0}\geq 1,\\ 0, & \text{if}\;R_{0}<1\;. \end{array} \end{array}$$ (4)

When *R*_0_ > 1, the establishment probability increases with the size of the inoculum *V*_*I*_(0). Indeed, for infection to succeed, only a single infectious virus particle among *V*_*I*_(0) needs to establish, so the more virus particles there are initially, the more likely it is that at least one establishes. Importantly, for a given *R*_0_, the virus establishes more easily when it has a low burst size *N*. Keeping the mean number of secondary cell infections *R*_0_ constant, a virus with a smaller burst size will have a larger infectivity *β* or smaller clearance *c*, which increases the first factor of *R*_0_ (Eq (2)). For the same number of virions to be produced at lower burst sizes, more cells need to be involved in viral production than for large burst sizes. This mitigates two risks incurred by the virus: the risk that it does not find a cell to infect before it is cleared, and the risk that the infected cell dies early by chance. Since more cells are involved in viral production for lower burst sizes, these risks are shared over all these virus-producing cells. This reduces the stochastic variance in viral production, which in turn results in a higher establishment probability.

### Prophylactic antiviral therapy blocks establishment of the virus

Next, we investigate the effect of prophylactic antiviral drug therapy on the establishment probability of the virus during the early phase of an infection. In particular, we examine drugs with four distinct modes of action.

1. Reducing the ability of the virus to infect cells *β*. This corresponds, for instance, to treatments that block viral entry, e.g. a neutralizing antibody (given as a drug) that binds to the spike glycoprotein [49].
2. Increasing the clearance of the virus *c*. This mode of action models drugs such as monoclonal antibodies that may be non-neutralizing or neutralizing and bind to circulating virus particles and facilitate their clearance by phagocytic cells [50].
3. Reducing viral production *p*. This mechanism corresponds, for example, to nucleoside analogues that prevent viral RNA replication (favipiravir, remdesivir), or to protease inhibitors (lopinavir/ ritonavir) [17].
4. Increasing infected cell death *δ*. This would describe the effect of SARS-CoV-2 specific antibodies that bind to infected cells and induce antibody-dependent cellular cytoxicity or antibody-dependent cellular phagocytosis. It would also model immunomodulatory drugs that stimulate cell-mediated immune responses, or immunotoxins such as antibody toxin conjugates that can directly kill cells [51].

We denote by *ε*_*β*_, *ε*_*c*_, *ε*_*p*_ and *ε*_*δ*_ the efficacies of the antiviral drugs in targeting the viral infectivity, viral clearance, viral production and infected cell death, respectively. Their values range from 0 (no efficacy) to 1 (full suppression). We neglect variations in drug concentrations over time within the host and, to be conservative, assume a constant drug efficacy corresponding to the drug efficacy at the drug’s minimal concentration between doses.

#### Antiviral reducing viral infectivity

Antiviral drugs reducing viral infectivity *β* by the factor (1 − *ε*_*β*_) leave the burst size *N* unchanged, but reduce the basic reproductive number, *R*_0_, by a factor $1-f(\varepsilon_{\beta })=1-\frac{c\varepsilon_{\beta }}{c+(1-\varepsilon_{\beta })\beta T_{0}}$. If (1 − *f*(*ε*_*β*_)) × *R*_0_ ≥ 1, the establishment probability changes to:

$$\begin{array}{c} \varphi_{\beta }=1-{\left(1-\frac{(1-f(\varepsilon_{\beta }))R_{0}-1}{\eta N}\right)}^{V_{I}\left(0\right)}. \end{array}$$ (5)

If (1 − *f*(*ε*_*β*_)) × *R*_0_ is less than 1, the virus will almost surely go extinct and we have *φ*_*β*_ = 0.

**Fig 2:**
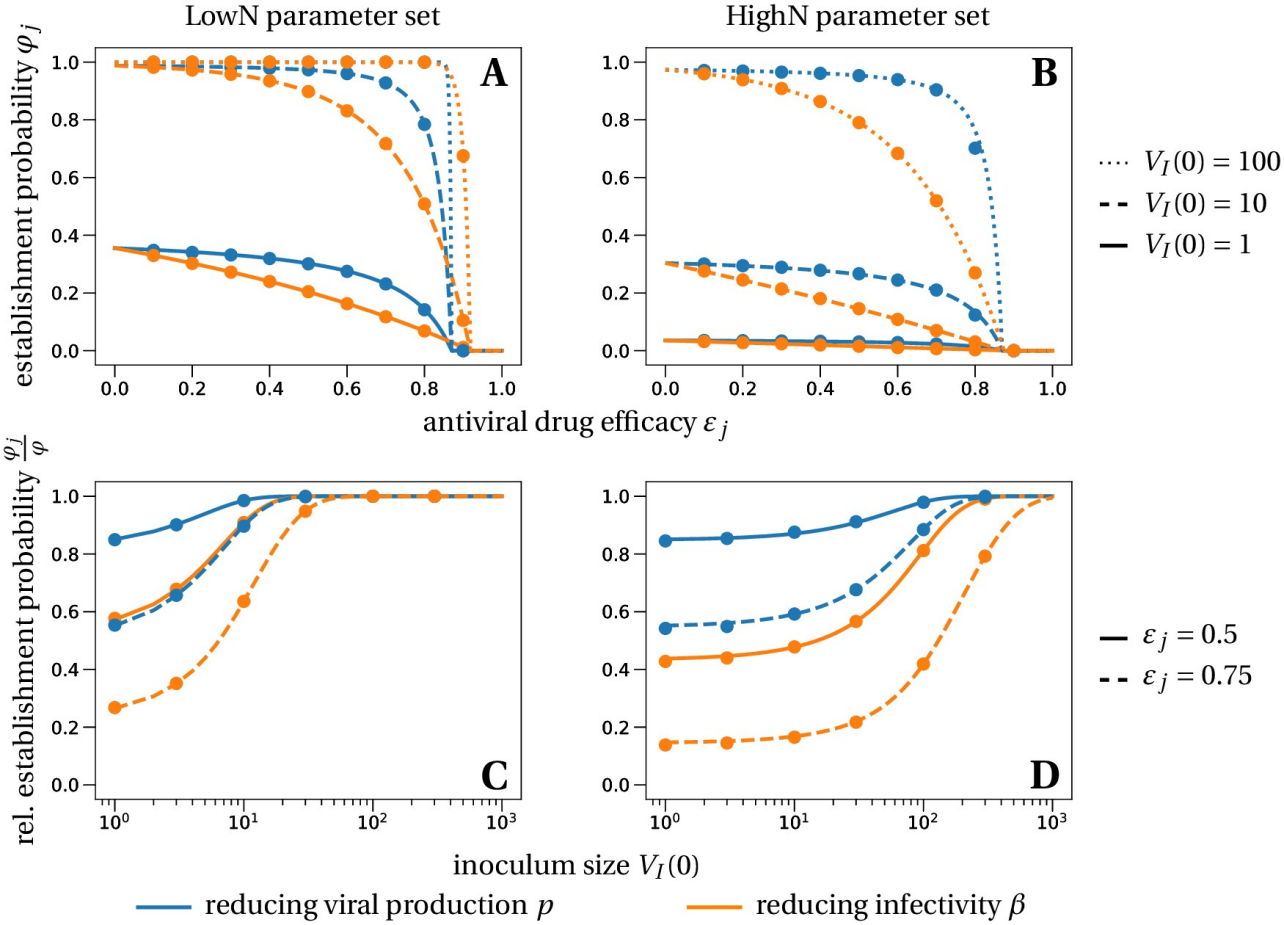
Establishment probability of a viral infection under prophylactic treatment with different antiviral drugs, efficacies *ε* and various inoculum sizes *V*_0_. The lines in panels A and B correspond to the theoretical establishment probability under the assumption that target cell numbers are constant, for the two modes of action (reducing viral infectivity equivalent to increasing clearance, Eq (5), in orange and reducing viral production equivalent to increasing cell death, Eq (6), in blue). The lines in the bottom panels represent the relative probability of establishment normalized by the establishment probability in the absence of treatment from Eq (4), i.e. *φ*_*j*_/*φ*. Dots are averages from 100, 000 individual-based simulations of the within-host model described in system (3), in which target cell numbers are allowed to vary. Parameter values are given in Table 2.

With a plausible inoculum size of 10 infectious virions [52, 53], we find that an efficacy (*ε*_*β*_) of 81% (LowN parameter set) is necessary to reduce the establishment probability of a viral infection by 50% compared to no treatment (see Fig 2A and 2C). Subsequently, when we mention the efficacy of an antiviral drug reducing viral infectivity, we always refer to *ε*_*β*_ and not *f*(*ε*_*β*_).

#### Antiviral increasing viral clearance

Antiviral drugs that increase the clearance rate *c* of extracellular virus particles reduce the average lifespan of a virus by a factor (1 − *ε*_*c*_). This changes the clearance parameter *c* by a factor 1/(1 − *ε*_*c*_).

With this definition of efficacy, we find that the reproductive number *R*_0_ is reduced by the same factor as obtained for a drug reducing infectivity: $(1-f(\varepsilon_{c}))=1-\frac{c\varepsilon_{c}}{c+(1-\varepsilon_{c})\beta T}$. Therefore, the establishment probabilities take the same form, so that *φ*_*c*_ = *φ*_*β*_. Consequently, we will reduce our analysis to antiviral drugs that reduce viral infectivity, keeping in mind that results for the establishment probability are equally valid for drugs increasing viral clearance.

#### Antiviral reducing viral production

Antiviral drugs reducing the viral production (parameter *p*) reduce the burst size *N* by a factor (1 − *ε*_*p*_). The basic reproductive number *R*_0_ is reduced by the same factor. If (1 − *ε*_*p*_) × *R*_0_ ≥ 1, such drugs alter the establishment probability to:

$$\begin{array}{c} \varphi_{p}=1-{\left(1-\frac{\left(1-\varepsilon_{p}\right)R_{0}-1}{(1-\varepsilon_{p})\eta N}\right)}^{V_{I}\left(0\right)}. \end{array}$$ (6)

A reduction of 50% of the establishment probability compared to no treatment can be achieved with an efficacy of 85% (LowN parameter set, *V*_*I*_(0) = 10). The efficacy needed is greater than that for antivirals targeting infectivity or viral clearance (81%) (see Fig 2A and 2C). Thus, for imperfect drugs that do not totally prevent establishment, drugs targeting infectivity (or clearance) are more efficient than those targeting viral production. This effect emerges from the stochastic dynamics and the reduction in viral production variance mentioned above: in the early phase, it is more important for the virus to infect many host cells than to ensure the production of a large number of virions. This insight might also affect the choice of antiviral drugs, depending on whether prophylaxis is taken pre- or post-exposure. In the case of pre-exposure, the scenario we mainly focus on and for which Eq (4) was derived, we would recommend to prioritize drugs that increase extracellular viral clearance or reduce viral infectivity. A neutralizing monoclonal antibody such as LY-CoV555 could do both. On the other hand, if prophylactic treatment is started post-exposure, e.g. a couple of hours after a potential between-host transmission event, the likelihood is high that cells are already infected. If cells are infected, the initial condition of our analysis is changed and drugs reducing viral production such as a SARS-CoV-2 polymerase inhibitor or protease inhibitor are more efficient in preventing the establishment of the virus than drugs targeting extracellular viral processes (clearance and target cell infection) in the LowN parameter set, cf. Section D in S1 Text.

#### Antiviral increasing infected cell death

Increasing the rate of death of infected cells *δ* by the factor 1/(1 − *ε*_*δ*_) reduces the average lifespan of an infected cell by a factor (1 − *ε*_*δ*_). This has the same effect on the burst size (and consequently on *R*_0_) as an antiviral drug reducing viral production, again due to our definition of efficacy. Therefore, the establishment probabilities are the same, *φ*_*p*_ = *φ*_*δ*_. In our analysis of establishment probabilities, we thus exclusively study antivirals affecting viral production.

### Critical efficacy

Above a critical treatment efficacy, the establishment of a viral infection is not possible. This is true for all modes of action and for high and low burst sizes (Fig 2). The critical efficacy does not depend on the initial inoculum size. It is given by the condition that the drug-modified *R*_0_ equals 1, e.g. (1 − *ε*_*p*_)*R*_0_ = 1 for drugs reducing viral production *p*. This corresponds to the deterministic threshold value for the viral population to grow. Computing the critical efficacies for both modes of action with Eqs (5) and (6), we find:

$$\begin{array}{c} {\tilde{\varepsilon }}_{p}\;=\;1-\frac{1}{R_{0}}\;<\;(1-\frac{1}{R_{0}})\frac{\eta N}{\eta N-1}\;=\;{\tilde{\varepsilon }}_{\beta }\;. \end{array}$$ (7)

They differ for the two modes of action because reducing infectivity does not proportionally reduce *R*_0_ (Eq (2)). Thus, drugs that reduce viral production result in a slightly lower critical efficacy, an effect that is small for a low burst size of infectious virions and not discernible with a high burst size of infectious virions (see intersections of the establishment probabilities with the x-axes in Fig 2A and 2B). For example, in the HighN parameter set, we find a critical efficacy of 87% for both types of drugs.

In summary, in the range where drugs cannot totally prevent infection, drugs that target viral infectivity reduce the probability of establishment more strongly; drugs that reduce viral production can totally prevent infection at slightly lower efficacy, but this difference is extremely small when burst sizes (of infectious virions) are large.

### Combination therapy

We analyze how the combination of two antiviral therapies could further impede establishment of the virus. We assume that two drugs that target different mechanisms of action lead to multiplicative effects on *R*_0_ (Bliss independence [54]). The establishment probability and critical efficacies for the two drugs can be computed in the same way as for single drug treatments.

For example, a combination of two drugs reducing viral production *p* and infectivity *β* changes the establishment probability to

$$\begin{array}{c} \varphi_{p,\beta }=1-{\left(1-\frac{\left(1-f\left(\varepsilon_{\beta }\right)\right)\left(1-\varepsilon_{p}\right)R_{0}-1}{(1-\varepsilon_{p})\eta N}\right)}^{V_{I}\left(0\right)}, \end{array}$$ (8)

if (1 − *f*(*ε*_*β*_))(1 − *ε*_*p*_)*R*_0_ ≥ 1.

The corresponding critical pair of efficacies that prevent viral infection entirely can be computed as before by solving

$$\begin{array}{c} \begin{array}{cc} & \;\left(1-f\left({\tilde{\varepsilon }}_{\beta }\right)\right)\left(1-{\tilde{\varepsilon }}_{p}\right)R_{0}=1, \end{array} \end{array}$$ (9)

By the arguments from above, we can replace *ε*_*β*_ by *ε*_*c*_ and *ε*_*p*_ by *ε*_*δ*_ without changing the results. Similar calculations allow us to derive the analogous quantities if we combine drugs targeting the same mechanism of action, e.g. altering *p* and *δ* or *c* and *β* at the same time. Our analysis would also carry over to combination of drugs which target the same parameter if they interact multiplicatively. For example, two drugs reducing viral infectivity *β* with efficacies *ε*_*β*,1_ and *ε*_*β*,2_, respectively, would reduce *R*_0_ by the factor (1 − *f*(*ε*_*β*,1_))(1 − *f*(*ε*_*β*,2_)), if they act independently.

**Fig 3:**
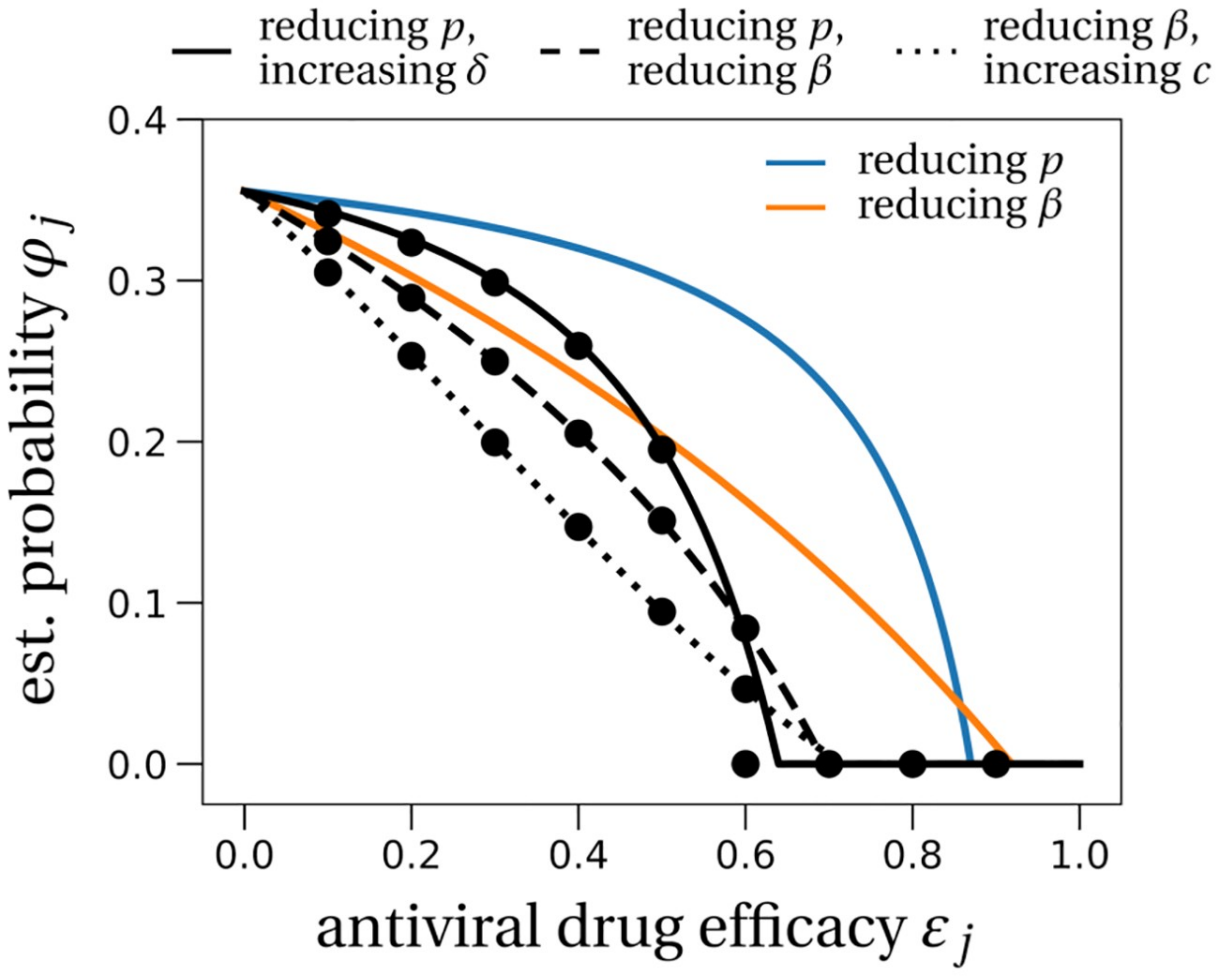
The effect of prophylactic combination therapy on the establishment probability. We compare different combination therapies (black lines) with the two single effect therapies (colored lines). The theoretical predictions for the combination therapies are variations of Eq (8), adapted to the specific pair of modes of action considered. We assume that both modes of action are suppressed with the same efficacy, shown on the x-axis as *ε*_*j*_. Dots are averages from 100, 000 stochastic simulations using the LowN parameter set and *V*_*I*_(0) = 1. In Section E in S1 Text, we study the effect of combination therapy in the HighN parameter set which overall leads to very similar results.

Using two drugs of limited efficacy in combination lead to large reductions in the establishment probability compared to the single drug or no treatment scenarios. For instance, two drugs with efficacies of 65% each may completely eliminate the risk of viral infection, depending on the combination used (LowN parameter set, *V*_*I*_(0) = 1, Fig 3). For comparison, a single drug with 65% efficacy can maximally reduce the establishment probability to ∼40% of the no-treatment establishment probability (see Fig 2A). We also find that, compared to the single drug cases, the critical efficacy is significantly reduced in all combinations studied.

In our analysis, we assumed that the drugs act independently (Bliss independence). This assumption may lead to an over- or underestimation of the establishment probability in case of antagonistic or synergistic drug interactions, respectively. These interactions are difficult to anticipate but were observed for HIV treatments [55].

### Time to detectable viral load and extinction time

Lastly, we quantify the timescales of viral establishment and extinction of infectious virus particles. If the virus establishes, we ask whether therapy slows down its spread within the host and investigate how long it takes for the infection to reach the polymerase chain reaction (PCR) test detection threshold. Conversely, if the viral infection does not establish, we examine how long it takes for antiviral therapy to clear all infectious virus and infected cells, which we define as the extinction time. We study all four modes of action: drugs that increase either the infected cell death rate *δ* or viral clearance *c*, and drugs reducing either viral production *p* or the infectivity *β*.

#### Time to detectable viral load

Even if antivirals are not efficacious enough to prevent establishment of the infection, could they still mitigate the infection? We study the effect of antiviral therapy on the time to reach a detectable viral load within the host. For example, the detection threshold in Young et al. [31] is at 10^1.84^ copies per mL. Assuming that the upper respiratory tract has a volume of about 30 mL [56], this corresponds to approximately 2, 000 virus particles.

**Fig 4:**
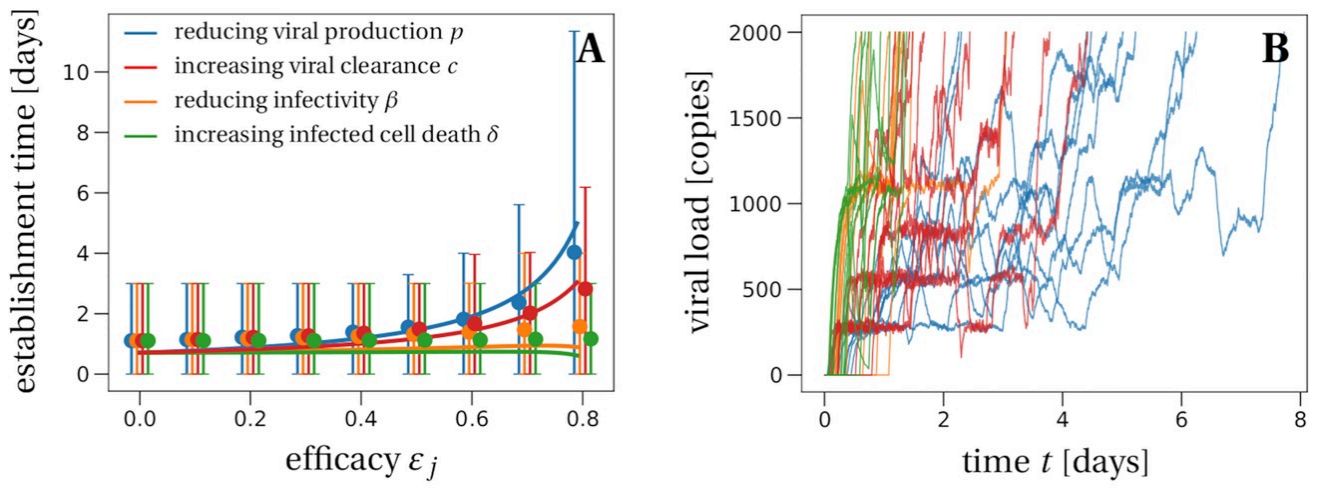
The mean time to reach a detectable viral load at the infection site. Panel A: Solid lines represent the theoretical prediction of the average time for the viral infection to reach 2, 000 virions (see Section F in S1 Text for details). We used the LowN parameter set to simulate 10, 000 stochastic simulations that reached a viral load of 2, 000 total virus particles when starting with an inoculum of *V*_*I*_(0) = 1. Dots are the average times calculated from these simulations, bars represent 90% of the simulated establishment times. We only consider efficacies below the critical efficacy (*ε*_*j*_ < 0.87, cf. Fig 2A) because above the critical efficacy infection is never established. Panel B: We plot 10 example trajectories that reach the detectable viral load for each of the four types of treatment (efficacy *ε*_*j*_ = 0.75). Under treatment that increases the infected cell death *δ* or reduces infectivity *β*, establishing trajectories reach the detectable viral load almost immediately. In contrast, drugs that directly affect the number of virus particles, i.e. clearance *c* or production *p*, allow for trajectories that fluctuate much more, explaining the larger average detection times and the larger variation of detection times for these scenarios.

In our model without treatment, the viral population size reaches 2, 000 within one day (see the leftmost data point in Fig 4). If establishment is likely, it is best to take antiviral drugs reducing the viral production *p* to delay the establishment of a viral infection as long as possible. This would reduce the peak viral load [19, 21], which is presumably correlated with the severity of SARS-CoV-2 infection [57]. The time to reach a detectable viral load depends on the growth rate of the viral population, which is to the leading order $\left(R_{0}-1\right)/(\frac{1}{c+\beta T_{0}}+\frac{1}{k}+\frac{1}{\delta })$ (see Section F in S1 Text for a derivation). The denominator is the average duration of a virus life cycle given by the sum of the phase when virions are in the medium, the eclipse phase of infected cells, and the phase during which infected cells produce virions until their death.

Importantly, the time to reach a detectable viral load is the earliest time when a patient can be tested to determine if therapy succeeded or failed to prevent infection. That time can be increased up to 4 days for drugs inhibiting viral production *p* (blue line in Fig 4), but there is significant variation with values ranging from smaller than one day to more than 10 days. Drugs reducing the infectivity *β* or increasing the infected cell death rate *δ* do not delay the establishment time. Drugs promoting viral clearance *c* increase the establishment time less than drugs decreasing the viral production rate *p*. As a brief explanation, when drugs target the infectivity or cell death, establishment occurs rapidly by full bursts of just two infected cells, which is enough to reach the detection threshold; when drugs target viral clearance or viral production, establishment may involve many more infected cells and occur slowly (Section F.2 in S1 Text).

#### Extinction time of infectious virus particles

Given that the infection does not establish, extinction of the within-host population of infectious virus particles typically happens within a day (in the HighN parameter set) to up to a week (in the LowN parameter set) depending on the drug’s mode of action (Table 3). We find that antiviral drugs that either reduce viral infectivity *β* or increase the infected cell death rate *δ* show comparably small extinction times (Table 3). The extinction time is useful to determine the number of days a potentially infected person should take antiviral medication post-exposure.

**Table 3:** Establishment probabilities (*φ*), times to detection (*T*_detect_) and extinction time (*T*_ext_) statistics for various sets of antiviral treatment.

*ε*_*j*_	Therapy		LowN parameter set		HighN parameter set	
			*V*_0_ = 1	*V*_0_ = 10	*V*_0_ = 1	*V*_0_ = 10
0	no treatment	*φ* *T*_detect_ *T*_ext_	36% 1 (0.5, 1.5) 0 (0, 0)	99% 0.5 (0, 0.5) 1 (0, 1.5)	4% 0.5 (0, 1) 0 (0, 0)	30% 0 (0, 0.5) 0.5 (0, 0.5)
0.75	reducing *p*	*φ* *T*_detect_ *T*_ext_	20% 4 (2, 9) 0 (0, 2)	89% 2 (0.5, 6.5) 2.5 (1, 6)	2% 0.5 (0, 1) 0 (0, 0)	18% 0.5 (0, 1) 0.5 (0, 2)
	increasing *δ*	*φ* *T*_detect_ *T*_ext_	20% 1 (0.5, 2) 0 (0, 1.5)	89% 0.5 (0, 1) 1.5 (1, 3)	2% 0.5 (0, 1) 0 (0, 0)	18% 0 (0, 0.5) 0.5 (0, 1.5)
	reducing *β*	*φ* *T*_detect_ *T*_ext_	9% 1 (0.5, 2.5) 0 (0, 0.5)	63% 0.5 (0.5, 2) 0.5 (0, 2.5)	1% 0.5 (0, 1) 0 (0, 0)	5% 0.5 (0, 1) 0.5 (0, 0.5)
	increasing *c*	*φ* *T*_detect_ *T*_ext_	9% 2.5 (1.5, 5.5) 0 (0, 0)	63% 2 (1, 5) 0 (0, 2)	1% 0 (0, 0.5) 0 (0, 0)	5% 0 (0, 0.5) 0 (0, 0)
0.9	reducing *p*	*φ* *T*_detect_ *T*_ext_	0% – 0 (0, 5)	0% – 7 (2.5, 19)	0% – 0 (0, 0.5)	0% – 0.5 (0, 5)
	increasing *δ*	*φ* *T*_detect_ *T*_ext_	0% – 0 (0, 2)	0% – 2.5 (1, 5)	0% – 0.5 (0, 1)	0% – 0.5 (0, 2)
	reducing *β*	*φ* *T*_detect_ *T*_ext_	1% 1.5 (0.5, 3.5) 0 (0, 0.5)	11% 1 (0.5, 3) 0.5 (0, 6)	0% – 0 (0, 0)	0% – 0.5 (0, 0.5)
	increasing *c*	*φ* *T*_detect_ *T*_ext_	1% 12 (5.5, 29) 0 (0, 0)	11% 12 (5, 28) 0 (0, 30)	0% – 0 (0, 0)	0% – 0 (0, 0)

The first value in each cell gives the establishment probability, the second value denotes the median time to detection (days), the numbers in brackets are the 10 and 90-percentiles of the time to detection distribution (days), and the last line gives the median time to extinction (days), conditioned on non-establishment of the infection, with the 10 and 90-percentiles in brackets. The detection threshold is set to 2, 000 virus particles. All times are rounded to half-day values if below 5 days, and to days if above. Missing values, denoted by dashes, are explained by the viral population not establishing. All results are estimated from 100, 000 stochastic simulations for the establishment probability and 10, 000 stochastic trajectories for the extinction and establishment times.

## Discussion

We have investigated the effect of prophylaxis with antiviral treatments including monoclonal antibodies on the viral dynamics of SARS-CoV-2. Using a stochastic model of within-host SARS-CoV-2 dynamics whose structure and parameters are informed by clinical data [19, 20], we showed that in principle a combination of two drugs each with efficacy between 60% and 70% will almost certainly prevent infection (Fig 3). For single drug treatment, we find that even intermediate efficacies can block infection, most efficiently with drugs reducing infectivity *β*, or otherwise delay the within-host establishment of the viral infection for drugs reducing viral production *p* or increasing viral clearance *c* (Fig 4). More generally, our stochastic model for the early phase of virus establishment within a host could be used to study the impact of prophylactic treatment on viral infections whose dynamics can be captured by the deterministic model in Eq (1).

This model makes several important assumptions. First, it encompasses a simplified version of the innate immune response. Effects of this type of immune reaction are embedded in the parameter values of the model. For example, an early innate response, if not effectively subverted by the virus, might put some target cells into an antiviral state where they are refractory to infection, thus effectively reducing *β* [29], or it could reduce the viral production rate *p* [58]. We neglect a potential adaptive immune response against the virus because we are interested in the early stages of the infection, before the immune system develops a specific response to the viral infection. A specific immune response may in later stages enhance the ability of the body to eliminate the virus. Models that explicitly include both types of immune responses have been shown to better fit the patient data from ref. [31] when compared to models without any immune response (based on the Akaike information criterion) [21]. Our estimates of the drug efficacies needed to prevent establishment of infection are therefore conservative and in reality may be overestimates. Even if the drugs being used do not have efficacies high enough to prevent infection on their own, they can lengthen the time needed to establish infection and hence allow the immune response to develop and assist in the clearance of the virus. Our model also includes the removal of virus particles due to cell infections (term −*βV*_*I*_ *T* in Eq (1)), a process typically neglected in deterministic models of virus dynamics, e.g. [20, 21, 59, 60]. In our mechanistic approach to model virus dynamics, this term is necessary to correctly describe the early dynamics of a viral infection while the number of infectious virus particles is still low. If we were to neglect loss of infectious virus particles due to cell infections, a single virus particle could potentially infect multiple target cells. This is problematic not only in the stochastic simulations, but also in the computation of the establishment probability of a viral infection. Lastly, we focus on the early phase of the infection in the upper respiratory tract, and neglect other compartments that may be more favorable to viral multiplication. For example, the number of virions in the sputum is (on average) 10 to 100 fold higher than in throat swabs [38]. The upper respiratory tract may allow a small amount of virus to enter the lower respiratory tract. It has also been observed in hamsters that the type of contact (airborne vs. fomite) affects the establishment probability and disease severity [61]. In future work, it would be interesting to explore the impact of this spatial structure and type of contact on viral dynamics and establishment probability.

Our results on critical efficacy, shown in Figs 2 and 3, do not depend on the viral inoculum size and are very similar for low and high burst sizes. However, they strongly depend on the within-host basic reproductive number which we estimated at *R*_0_ = 7.69. This basic reproductive number was estimated from time series of viral load in nasopharyngeal swabs in 13 infected patients [19, 31] and is consistent with the mean peak viral load observed in multiple studies (Table 1). Still, there is substantial inter-individual heterogeneity in incubation time, observed peak viral timing and load [39]. A shorter time to the viral load peak or a higher viral load peak would result in higher estimates of *R*_0_ (Fig 1B). Yet, our qualitative findings on the effectiveness of prophylactic therapy remain valid under these variations of *R*_0_. Of course, the quantitative predictions, which depend on *R*_0_, change. Considering the current uncertainty in the basic reproductive number and burst size, we developed an interactive application to compute and visualize the establishment probability and deterministic dynamics as a function of parameters. This application can be used to update our results as our knowledge of within-host dynamics and treatment efficacies progresses (it can be accessed by following the instructions on github.com/pczuppon/virus_establishment/tree/master/shiny).

The critical efficacy above which infection is entirely prevented is the efficacy at which the within-host basic reproductive number, adjusted for the antiviral drug under consideration, passes below 1. The value of this critical efficacy could readily be obtained in a deterministic model. This theoretical value can probably be translated directly to in-vitro experiments. Yet, a translation from measured in-vitro efficacies to in-vivo application is more challenging as studies in the context of HIV have shown: drug efficacies obtained from in-vitro experiments typically overestimate the actual in-vivo efficacy [62, 63]. Still, our stochastic framework gives several new additional insights into the probability of establishment. Importantly, below the critical efficacy, viral establishment is not certain. The establishment probability increases with the size of the initial inoculum (Fig 2). The number of infectious virions of seasonal coronavirus in droplets and aerosol particles exhaled during 30 minutes could be in the range of 1 to 10 [52]. For SARS-CoV-2, inoculum sizes ranging from less than 10 [53] to the order of 1,000 infectious virus particles [64] have been estimated. Assuming the inoculum of infectious virus particles to be of the order of 10, in most cases the establishment of a viral infection is not ensured even with low-efficacy drugs. For efficacies below the critical efficacy, drugs reducing infectivity or increasing viral clearance reduce the establishment probability the most. Examples for this type of drug include monoclonal neutralizing antibodies that recently have shown promising results for treatment and prophylaxis of SARS-CoV-2 [65]. In contrast, drugs reducing viral production need to be close to critical efficacy to cause a marked reduction on the probability of establishment (Figs 2 and 3). Several studies are underway to assess the prophylactic potential of repurposed drugs blocking viral production, such as lopinavir, favipiravir or remdesivir, but there is no clear demonstration that these drugs can achieve clinically relevant antiviral efficacy [66–68].

Similar theoretical results have been obtained for HIV antiviral prophylactic treatments [69]. If initially there is one infectious HIV particle, drugs that target viral production within cells are less successful in inhibiting infection than drugs that reduce viral infection of target cells, cf. Fig 2A in [69]. However, if the virus has already infected a cell, the difference between the two drug types vanishes, i.e., both modes of action equally reduce the establishment of an infection (Fig 2B and 2C in [69]). In contrast, with our model we find that if there is initially one infected cell, establishment of a viral infection is suppressed more strongly by drugs that reduce viral production than by those reducing infection of target cells (Section D in S1 Text). This difference most likely arises due to the different burst sizes of infectious virus particles assumed in the two models. Here, we assume that the burst size is around 20 infectious virus particles, computed by *η* × *N*. In contrast, the HIV model studied in [69] assumes a burst size of 670. Indeed, increasing the burst size in our model, the HighN parameter set, recovers the result found in [69], i.e., the two different drug types affect the establishment probability equally.

Lastly, we observe that given that extinction occurs the time to extinction is largely independent of the drug’s mode of action and typically occurs within a day (Table 3). In contrast, we find a relatively strong dependence of the time to detection of an infection on the mode of action of the antiviral drug. The time to detection also strongly depends on the burst size which varies substantially depending on the assumed fraction of infectious virus particles produced, *η*. For example, a lower fraction than considered here in the main text will result in a higher burst size for a fixed value of *R*_0_ (Section G.2 in S1 Text) and consequently in a lower time to detection. If the delay between exposure and therapy, as well as the efficacy of the available drugs, are such that establishment of the viral infection is almost certain, antiviral drugs that reduce viral production (parameter *p*) will slow down the exponential growth and flatten the within-host epidemic curve the most (Fig 4). Repurposed antiviral drugs reducing viral production were recently proposed as good drug candidates against SARS-CoV-2 [18]. This prolonged period at low viral loads could give the immune system the necessary time to activate a specific response to the virus and develop temporary host-immunity against SARS-CoV-2. This might be especially important in groups that are frequently exposed to the virus, e.g. health care workers. Still, since reducing the infection probability itself is the primary goal, drugs reducing the infectivity of virus (parameters *β* and *c*) should be favored over drugs reducing viral production (parameters *p* and *δ*) because of their stronger effect on the establishment probability (Fig 2).

## Conclusion

Clinical trials are underway to test the efficacy of several antiviral drugs [16, 17, 66, 70, 71], either as a curative treatment or as a prevention. The efficacy of repurposed drugs is in a 20-70% range [19], but better antiviral drugs might be available soon. With our model, the individual values of *R*_0_ for the 13 untreated patients from ref. [31] range from 1.58 to 15.47 (Table B in S1 Text) which approximately translates to critical efficacies between 37% and 94% in the case of drugs reducing viral production, ${\tilde{\varepsilon }}_{p}$ (Eq (7)). An interactive tool has been made available to update the prediction of critical efficacies with refined parameter estimates that may come from large dataset obtained in the different target populations where prophylaxis may be relevant (such as health care workers or high-risk individuals). Given the current knowledge of SARS-CoV-2 viral dynamics, our model predicts that prophylactic antiviral therapy can block (or at least delay) a viral infection, could be administered to people at risk such as health care workers, and alleviate the burden on the healthcare systems caused by the SARS-CoV-2 pandemic.

### Methods

#### Simulations

The individual based simulations are coded in C++ using the standard stochastic simulation algorithm for the reactions described in system (3).

Estimates for the establishment probabilities, depicted by dots in the subsequent figures, are averages of 100, 000 independent runs. Establishment was considered successful when the population size of infectious virions was at least 500. Estimates for the time to reach a detectable viral load are obtained from 10, 000 simulations where the sum of infectious and non-infectious virus particles exceeded 2, 000 copies.

The code and the data to generate the figures are available at: github.com/pczuppon/virus_establishment.

## Supporting information

S1 Text Theoretical derivations and additional analyses. (PDF)
